# Recurring hyperammonemic encephalopathy induced by bacteria usually not producing urease

**DOI:** 10.1186/1756-0500-7-324

**Published:** 2014-05-31

**Authors:** Christian Cordano, Elisabetta Traverso, Valentina Calabrò, Chiara Borzone, Silvia Stara, Roberta Marchese, Lucio Marinelli

**Affiliations:** 1 Institute of Neurology, Department of Neuroscience, Rehabilitation, Ophthalmology, Genetics, Maternal and Child Health, University of Genova, Largo Daneo 3, 16132 Genova, Italy

**Keywords:** Hyperammonemic encephalopathy, Urease, Neurogenic bladder, Urinary retention, Urea-splitting bacteria, Urinary tract infections

## Abstract

**Background:**

Hyperammonemic encephalopathy may occur when urease-positive bacteria in the urinary tract produce ammonium which directly enters systemic circulation. Predisposing conditions such as a neurogenic bladder can increase both urinary tract infection and urine stagnation.

**Case presentation:**

We describe the case of a 66 years old woman with a neurogenic bladder who twice developed hyperammonemic encephalopathy following urinary tract infection. During the second episode Escherichia coli and Enterococcus faecalis have been isolated in the urine. The neurologic examination showed psychomotor slowing, weak photomotor reflex, nystagmus in the lateral gaze and asterixis. The EEG showed triphasic waves which disappeared along with clinical recovery.

**Conclusion:**

Escherichia coli and Enterococcus faecalis are commonly considered urease-negative bacteria. Although frequently involved in urinary tract infections, their role in causing hyperammonemic encephalopathy have not been previously reported. Moreover, despite only one case with a neurogenic bladder have been described so far, our is the first patient with reoccurring hyperammonemic encephalopathy secondary to urinary tract infections.

## Background

The occurrence of hyperammonemic encephalopathy is frequently related to liver disease and portal hypertension, where hepatic detoxification process is impaired. In patients with urinary divertion, vesico-colonic fistula or congenital anomalies affecting urinary tract function, ammonium may bypass portal circulation draining directly into the hypogastric veins and inferior vena cava. During urinary tract infections (UTI), urea splitting organisms may determine ammonium production primarily within the urinary system. There are rare conditions when the urine remains into a dilated bladder long enough to allow ammonium reach systemic circulation: this can be due to large diverticula [1], a neurogenic bladder [2] or isolated urinary retention [3,4].

We report the case of a patient with a neurogenic bladder who presented two episodes of hyperammonemic encephalopathy three years apart as a result of UTI. During the last episode, urine culture revealed Escherichia coli and Enterococcus faecalis which are considered urease-negative bacteria.

## Case presentation

**Figure 1:**
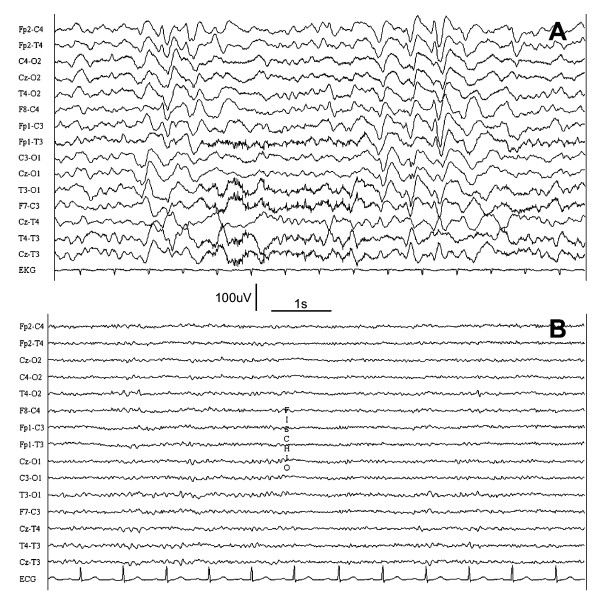
**EEG recording showing frequent triphasic waves during hyperammonemic encephalopathy (A) and significant improvement after recovery (B).** TC 0.01 s, HF 300 Hz.

A 66-years-old female, with a history of urinary retention and recurrent UTI, became progressively somnolent during one week, complaining at the same time dysuria and bladder pain, for which she had been assuming trimethoprim-sulfamethoxazole. She was conducted to our department after the execution of a brain CT (no alterations) and the detection of hyperammonemia (89 μmol/L) in the emergency room. The neurologic examination showed psychomotor slowing, weak photomotor reflex, nystagmus in the lateral gaze and asterixis. Her past medical history highlighted that, three years before, she had experienced a similar event (altered state of consciousness and negative myoclonus in concurrence with a UTI, resolved with antibiotic treatment). She underwent laboratory blood and urine tests, which showed increased pH (8.0) and a urinary tract infection caused by Escherichia coli and Enterococcus faecalis along with mild increase of serum creatinine, but without impairment of hepatic function; EEG showed alterations consistent with toxic metabolic encephalopathy (Figure 1). Underlying metabolic causes of hyperammonaemic encephalopathy (liver function tests, organic acids, amino acids, urea cycle defect and organic acidaemia) have been excluded. She was treated with ciprofloxacin, with resolution of the neurologic and electroencephalographic abnormalities in a few days.

## Discussion

Hyperammonemic encephalopathy occurs when hyperammonemia causes glutamine-mediated effects on the brain, such as astrocytic swelling, cerebral edema, and raised intracranial pressure [5]. An increase of ammonia levels in blood has been described in different clinical conditions, such as hepatic failure [6], urea cycle disorders, and, rarely, after therapeutic procedures on the urinary tract [1,5,7,8]. In fact, hyperammonemia may be provoked by either a failure of the ammonia metabolism by the hepatic mitochondria, with consequent impairment of the urea cycle, or an increase in the amount of ammonia produced by urease-positive pathogens. Since urease catalyzes the hydrolysis of urea to produce ammonia, the action of urea-splitting organisms result in an increased production of ammonia and its absorption in the circulation. Urinary tract infection by Klebsiella oxytoca, Klebsiella pneumoniae, Proteus sp, Corynebacterium sp and Staphylococcus aureus (known as urease-positive bacteria) have been reported to be involved in hyperammonemic encephalopathy in children and adults [3,4,7,9,10]. Our patient developed a UTI by two opportunistic organisms, Escherichia coli and Enterococcus faecalis, which are commonly not considered urease producers and have not been previously reported causing hyperammonemic encephalopathy. We hypothesize that at least one of these bacteria developed the ability to produce urease, although this could not have been directly confirmed by a laboratory test.

Our patient has been suffering from urinary retention for many years because of an idiopathic neurogenic bladder. Urinary retention not only predisposed her to the infection, but also increased the absorption of ammonia from the leftover urine through the bladder walls [3].

Although hyperammonemic encephalopathy determines a similar clinical and EEG picture independently of the cause (UTI or hepatic failure), the urinary origin is likely to result in a faster (days or weeks) onset of the symptoms, compared with the chronic and peculiar clinical picture of patients with hepatic dysfunction.

To our knowledge this is the first described case where urinary retention was associated with urinary tract infection and recurring hyperammonemic encephalopathy. Moreover no previous cases have been reported where urinary tract infection by Escherichia coli and Enterococcus faecalis caused hyperammonemia.

## Conclusions

The present case focuses on urinary tract infection related to neurogenic bladder as a potential cause of hyperammonemia, which nevertheless can lead to metabolic encephalopathy. It is important to underline that bacteria usually considered urease-negative may be related to ammonium production within the urinary tract.

## Consent

Written informed consent was obtained from the patient for publication of this Case Report and any accompanying images. A copy of the written consent is available for review by the Editor-in-Chief of this journal.

## Abbreviations

UTI: Urinary tract infection; CT: Computed tomography; EEG: Electroencephalogram.

## Competing interests

The authors declare that they have no competing interests.

## Authors’ contributions

All the authors have read and approved the manuscript. In addition all authors have contributed in the composition, write-up, and review of the manuscript.
